# Gold nanoparticles as cell regulators: beneficial effects of gold nanoparticles on the metabolic profile of mice with pre-existing obesity

**DOI:** 10.1186/s12951-018-0414-6

**Published:** 2018-11-03

**Authors:** Hui Chen, Jane P. M. Ng, David P. Bishop, Bruce K. Milthorpe, Stella M. Valenzuela

**Affiliations:** 1 School of Life Sciences, Faculty of Science, University of Technology Sydney, Sydney, Australia; 2 Centre for Health Technologies, University of Technology Sydney, Sydney, Australia; 3 School of Mathematical and Physical Sciences, University of Technology Sydney, Sydney, Australia

**Keywords:** Gold nanoparticles, Inflammation, Liver, Obesity, Metabolism

## Abstract

**Background:**

We have previously shown that intraperitoneal injection of gold nanoparticles (AuNPs, 20–30 nm) into mice, decreases high-fat diet (HFD) induced weight gain and glucose intolerance, via suppression of inflammatory responses in both fat and liver tissues. This study investigates whether AuNPs provide similar benefit to mice with pre-existing obesity. Male C57BL/6 mice were fed a HFD for 15 weeks. AuNPs (OB-EAu 0.0785 μg/g/day, OB-LAu 0.785 μg/g/day, OB-HAu7.85 μg/g/day, ip) were administered to subgroups of HFD-fed mice over the last 5 weeks. Control group was fed standard chow and administered vehicle injection.

**Results:**

Only the OB-LAu group demonstrated significant weight loss (12%), while all AuNP treated groups showed improved glycaemic control and reduced blood lipid levels. In the fat tissue, mRNA expression of pro-inflammatory markers were unchanged following AuNP treatment, while glucose and lipid metabolic markers were improved in OB-LAu and OB-HAu mice. In the liver, AuNP treatment downregulated inflammatory markers and improved lipid metabolic markers, with marked effects in OB-EAu and OB-LAu groups.

**Conclusions:**

AuNP treatment can improve glucose and fat metabolism in mice with long-term obesity, however weight loss was only observed in a single specific dose regime. AuNP therapy is a promising new technology for managing metabolic disorders in the obese.

**Electronic supplementary material:**

The online version of this article (10.1186/s12951-018-0414-6) contains supplementary material, which is available to authorized users.

## Background

The global obesity pandemic is mainly driven by life style, including the lack of physical exercise and overconsumption of diets that are high in fat and simple carbohydrates [1]. With the handful of current available anti-obesity drug interventions, patients usually regain some or all of the weight that was originally lost after discontinuation of treatment [2, 3]. Obesity treatment through bariatric surgery such as gastrointestinal Roux-en-Y bypass, has been shown to be the only effective long-term weight loss strategy [4, 5], with significantly improved glucose regulation and the perception of both hunger and satiety following such surgery [6, 7]. This procedure however is often reserved for patients that are morbidly obese, as a last resort due to its complications which frequently require further follow up surgery [7, 8]. After the surgery, the risk of obesity comorbidity and mortality are significantly reduced, particularly in relation to diabetes and cardiovascular diseases [9, 10]. However, such procedures are very costly and not freely available to most of the overweight and obese individuals. Thus, a more widely available and cost-effective treatment option is needed.

Chronic obesity is a state of chronic low-grade inflammation. In the adipose tissue, the recruitment and infiltration of circulating macrophages are the key to excess storage of lipids and drive pro-inflammatory responses via their secretion of pro-inflammatory cytokines, e.g. TNFα and IL-6 [11, 12]. The latter also has significant implications in insulin resistance in multiple organs, including both fat tissue and liver. As we have previously published, mice placed on a high fat diet (HFD) resulting in obesity, have increased macrophage activity along with insulin resistance, glucose intolerance, hyperlipidaemia, and liver steatosis [13].

Liver steatosis is ectopic lipid accumulation in the liver which is a common asymptomatic liver condition found in most obese individuals with central adiposity and insulin resistance [14, 15]. The infiltration of fat in non-adipose organs is promoted by the influx of free fatty acid from the diet and increased lipolysis in the fat tissue due to excessive fat accumulation during weight gain, and decreased fatty acid β-oxidation, leading to increased de novo lipogenesis and triglyceride over accumulation [16, 17]. Liver steatosis can also induce inflammatory responses through the activation of Kupffer cells (liver resident macrophages) and production of pro-inflammatory TNFα, similar to the above mentioned changes in the adipose tissue [18, 19]. Studies have shown that elevated hepatic TNFα signalling is essential for the progression of non-alcoholic fatty liver disease and liver fibrotic changes [20, 21].

Gold nanoparticles (AuNPs) are now well recognised for their useful properties of biocompatibility, low cytotoxicity and cell regulatory effects which can be exploited for medical prophylactic and therapeutic purposes [22]. We have undertaken a series of studies, including the current one, examining their suitability as agents for regulation of metabolic processes and immune cell activities [23, 24]. This is the third in a three part study looking at the effects of AuNPs injected intraperitoneally into mice. The first study used normal mice [24], the second study involved treatment of mice with AuNPs while consuming a HFD [23] and in this study, the treatment was of obese mice. Previously, we demonstrated the beneficial effects of unmodified spherical gold nanoparticles administered to mice simultaneously fed a HFD, in order to slow down excessive weight gain, as well as reduce pro-inflammatory responses and improve glucose tolerance and blood lipid profiles [23]. The effect seen was prominent across two dose regimes. In the current study we have applied the same AuNP dose regime [23, 24] but have used mice with existing obesity due to long-term high fat consumption. In addition, we also included a group of obese mice treated with extremely low dose of AuNP to investigate the lowest treatment threshold.

## Results

### Characterization of AuNPs

AuNPs prepared via the citrate-reduction method yielded a colloidal stable suspension. The dynamic light scattering measurement showed a narrow distribution of size in water with hydrodynamic diameter of between 17 and 30 nm with a mean of 27.3 ± 0.5 nm in size (Additional file 1: Figure S1) and surface zeta potential of − 37.0 ± 1.1 mV for the as-synthesized or citrate-coated AuNPs. The as-synthesized AuNPs dispersed in water showed characteristic surface plasmon resonance peak of 520 nm as determined from UV–VIS absorption spectrum (Additional file 1: Figure S2). HR-SEM at high magnifications showed monodispersed spherical shaped AuNPs, with an average measured AuNPs core diameter of 17.6 ± 0.3 nm in size (Additional file 1: Figure S3).

The centrifuge-purified AuNPs showed a shifted surface zeta potential of − 30.4 ± 0.7 mV and a red-shift in surface plasmon resonance peak to 518 nm due to the removal of excess negative charge of citrate capping (Additional file 1: Figure S2). The size of centrifuge-purified AuNPs was not changed by removal of excess citrate ions with a measured particle core size of 17.0 ± 0.3 nm determined using HR-SEM (Additional file 1: Figure S3).

### Effects of HFD consumption

#### Anthropometric and metabolic parameters

HFD feeding increased the body weight by 47% at 10 weeks (P < 0.05, all HFD groups vs Chow, Table 1) and the body weights of both OB and Chow groups plateaued afterwards (Table 1, Additional file 1: Figure S4). At 15 weeks, the OB group had 53% greater body weight and 24% greater energy consumption than the Chow group (P < 0.05, Table 1). Fat, and liver masses in the OB group were also significantly greater than the Chow group, with the retroperitoneal, epididymal and mesenteric fat masses 5, 2.5 and 4 times greater than the Chow group, respectively (P < 0.05, OB vs Chow, Table 1). The differences remained significant following standardisation with the body weight (P < 0.05, OB vs Chow).

**Table 1:** Effects of HFD and AuNP treatment on anthropometric parameters

	Chow	OB	OB-EAu	OB-LAu	OB-HAu
Body weight initial (g)	23.0 ± 0.3	22.9 ± 0.3	23.0 ± 0.3	23.0 ± 0.3	22.9 ± 0.4
Body weight at 10 weeks (g)	30.1 ± 0.6	44.2 ± 0.8*	44.2 ± 0.6*	44.7 ± 0.6*	44.3 ± 0.6*
Body weight at 15 weeks (g)	29.9 ± 0.5	45.6 ± 0.8*	43.0 ± 0.7*^,†^	40.0 ± 1.2*^,†^	44.6 ± 0.5*
Energy intake (kJ/day)	44.6 ± 1.0	55.7 ± 1.9*	60.6 ± 1.9*	51.6 ± 4.1*	59.0 ± 2.0*
Liver (g)	1.41 ± 0.04	2.85 ± 0.19*	2.64 ± 0.12*	2.10 ± 0.13*^,†^	2.66 ± 0.12*
Liver (%)	4.74 ± 0.13	6.20 ± 0.31*	6.10 ± 0.22*	5.20 ± 0.19^†^	5.94 ± 0.23*
Retroperitoneal fat (g)	0.14 ± 0.02	0.71 ± 0.04*	0.56 ± 0.04*^,†^	0.51 ± 0.05*^,†^	0.62 ± 0.02*
Retroperitoneal fat (%)	0.46 ± 0.06	1.56 ± 0.08*	1.30 ± 0.07*^,†^	1.23 ± 0.09*^,†^	1.38 ± 0.04*
Mesenteric fat (g)	0.43 ± 0.03	1.12 ± 0.04*	1.19 ± 0.07*	1.02 ± 0.09*	1.20 ± 0.04*
Mesenteric fat (%)	1.45 ± 0.08	2.44 ± 0.10*	2.76 ± 0.13*	2.53 ± 0.17*	2.69 ± 0.08*
Epididymal fat (g)	0.51 ± 0.04	2.08 ± 0.08*	2.43 ± 0.08*^,†^	1.81 ± 0.12*^,†^	2.25 ± 0.08*
Epididymal fat (%)	1.17 ± 0.12	4.57 ± 0.21*	5.66 ± 0.19^†^	4.54 ± 0.29*	5.05 ± 0.19*

Results are expressed as mean ± S.E.M. Data were analysed by one-way ANOVA followed by post hoc Bonferroni test

* P < 0.05 vs Chow; ^†^ P < 0.05 vs OB. n = 9–15

Blood glucose levels in the OB group during the intraperitoneal glucose tolerance test (IPGTT) was consistently higher than the Chow group at 15, 30, 60 and 90 min post glucose injection (P < 0.05, Additional file 1: Figure S5). The area under the curve (AUC) of the OB mice was 56% higher than the Chow group (P < 0.01, Table 2). Non-fasting plasma insulin was also significantly increased in the OB mice by 2.7 times (P < 0.05 vs Chow, Table 2).

**Table 2:** Effect of HFD and AuNP treatment on metabolic parameters

	Chow	OB	OB-EAu	OB-LAu	OB-HAu
Area under the curve (mM min)	1452 ± 42	2270 ± 210**	1812 ± 118*^,††^	1758 ± 99*^,††^	1945 ± 101**^,†^
Plasma insulin (ng/mL)	0.012 ± 0.001	0.032 ± 0.007*	0.039 ± 0.011*	0.028 ± 0.004	0.035 ± 0.007*
Plasma NEFA (mM)	2.07 ± 0.16	3.38 ± 0.31*	2.63 ± 0.21	2.56 ± 0.23^†^	2.83 ± 0.35*
Plasma triglyceride (mM)	0.62 ± 0.09	0.60 ± 0.10	0.37 ± 0.04	0.34 ± 0.05*	0.40 ± 0.06
Plasma HDL-C (mM)	1.42 ± 0.35	1.64 ± 0.22	3.40 ± 0.34**^,††^	2.79 ± 0.20**^,††^	3.00 ± 0.23**^,††^
Plasma AST (U/L)	7.45 ± 1.35	50.14 ± 9.41*	41.94 ± 2.87*	25.30 ± 5.17^†^	23.46 ± 2.70^†^
Plasma ALT (U/L)	6.40 ± 0.68	20.01 ± 3.18*	12.08 ± 2.90	23.85 ± 3.32*	25.35 ± 4.41*
Liver triglyceride (mM/mg tissue)	0.05 ± 0.01	0.60 ± 0.07*	0.55 ± 0.04*	0.58 ± 0.05*	0.44 ± 0.05*^,†^

Results are expressed as mean ± S.E.M. Data were analysed by one-way ANOVA followed by post hoc Bonferroni test

* P < 0.05, ** P < 0.01 vs Chow; ^†^ P < 0.05, ^††^ P < 0.01 vs OB. n = 4–15

Plasma non-esterified free fatty acids (NEFA) concentration was significantly elevated 63% by HFD consumption (P < 0.05 vs Chow) without significant changes in plasma triglyceride and high-density lipoprotein cholesterol (HDL-C) levels (Table 2). However, liver triglyceride concentration was significantly increased by 11-fold in the OB mice (P < 0.05 vs Chow, Table 2) indicating liver steatosis. Plasma alanine aminotransferase (ALT) and aspartate aminotransferase (AST) levels were also significantly elevated by 2.1-fold and 5.7-fold (P < 0.05, OB vs Chow, Table 2), respectively, suggesting some level of liver cell damage.

#### Inflammatory and metabolic markers

**Fig. 1:**
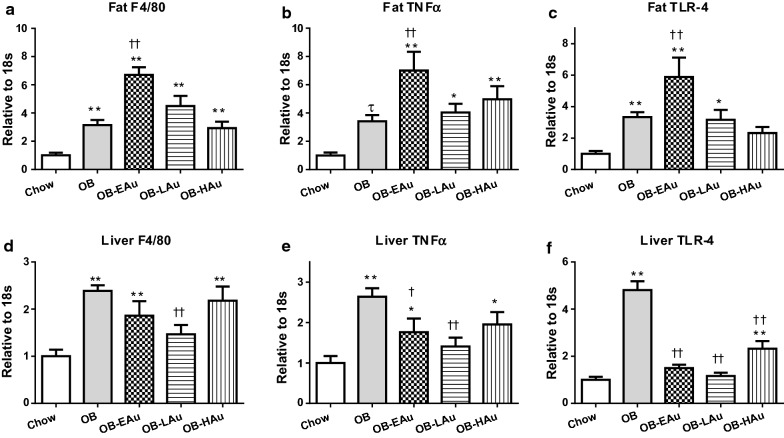
Effect of HFD and AuNP treatment on pro-inflammatory markers in abdominal fat and liver. mRNA expression of F4/80 (**a**, **d**), TNFα (**b**, **e**), and TLR-4 (**c**, **f**) in Chow, OB, OB-EAu, OB-LAu and OB-HAu mice at 15 weeks. Results are expressed as mean ± S.E.M. Data were analyzed by one-way ANOVA followed by post hoc Bonferroni test; *P < 0.05, **P < 0.01 vs Chow; ^†^P < 0.05, ^††^P < 0.01 vs OB. Data analyzed with conditional student *t* test followed by Welch correction, τ P < 0.05 vs Chow; n = 5–8

In the fat tissue, there was a three-fold increase in F4/80 (P < 0.01, Fig. 1a) mRNA expression in the OB group in comparison to the Chow group. TNFα and its upstream toll-like receptor (TLR)-4 mRNA were also significantly upregulated with an almost fourfold increase in the OB mice (P < 0.05 and P < 0.01 respectively) vs Chow fed mice (Fig. 1b, c). In the liver F4/80 mRNA expression was also more than doubled (P < 0.01 vs OB, Fig. 1d) by HFD consumption, with markedly increased TNFα and TLR-4 mRNA expression (both P < 0.001, OB vs Chow, Fig. 1e, f).

**Fig. 2:**
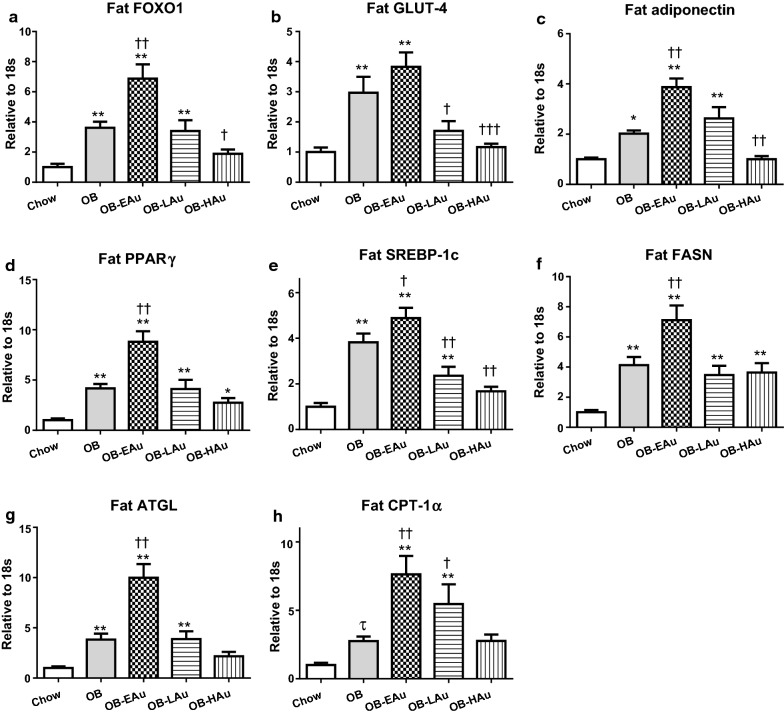
Effect of HFD and AuNP treatment on lipid and glucose metabolic markers in the fat. mRNA expression of FOX-O1 (**a**), GLUT-4 (**b**), adiponectin (**c**), PPARγ (**d**), SREBP-1c (**e**), FASN (**f**), ATGL (**g**), and CPT-1α (**h**) in Chow, OB, OB-EAu, OB-LAu and OB-HAu mice. Results are expressed as mean ± S.E.M. Data were analysed by one-way ANOVA followed by post hoc Bonferroni test. *P < 0.05, **P < 0.01 vs Chow; ^†^P < 0.05, ^††^P < 0.01 vs OB; Data analysed with conditional student *t* test followed by Welch correction, τ P < 0.05 vs Chow; n = 6–8

As shown in Fig. 2, long term HFD consumption by the OB group led to increased mRNA expression of all metabolic markers measured in the retroperitoneal adipose tissue, including glucose metabolic markers [forkhead box protein O1 (FOX-O1), glucose transporter (GLUT)-4, P < 0.01 vs Chow, Fig. 2a, b], markers related to insulin sensing [adiponection, P < 0.05, peroxisome proliferator-activated receptors (PPAR)γ P < 0.01 vs Chow, Fig. 2c, d], and lipid metabolic markers [sterol regulatory element-binding proteins (SREBP)-1c, fatty acid synthase (FASN), Adipose triglyceride lipase (ATGL) P < 0.01, carnitine palmitoyltransferase (CPT)-1α P < 0.05, vs Chow, Fig. 2e–h].

**Fig. 3:**
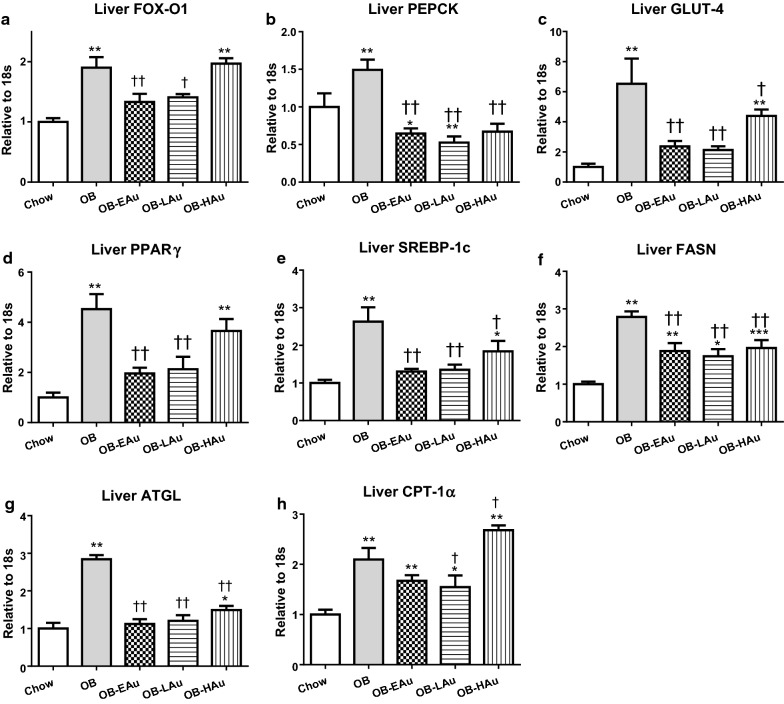
Effect of HFD and AuNP treatment on lipid and glucose metabolic markers in the liver. mRNA expression of FOX-O1 (**a**), PERCK (**b**), GLUT-4 (**c**), PPARγ (**d**), SREBP-1c (**e**), FASN (**f**), ATGL (**g**), and CPT-1α (**h**) in Chow, OB, OB-EAu, OB-LAu and OB-HAu mice. Results are expressed as mean ± S.E.M. Data were analysed by one-way ANOVA followed by post hoc Bonferroni test. *P < 0.05, **P < 0.01 vs Chow; ^†^P < 0.05, P < 0.01 vs OB; n = 5–8

Similarly, in the liver, glucose and lipid metabolic markers were also significantly increased in the OB mice (Fig. 3). mRNA expression of FOXO1, phosphoenolpyruvate carboxykinase (PEPCK) and GLUT-4 were increased by 2, 1.5 and sixfold respectively (P < 0.01 vs Chow, Fig. 3a–c), while PPARγ was significantly increased by 4.5-fold (P < 0.01 vs Chow, Fig. 3d). SREBP-1c, FASN, ATGL mRNA levels were all increased threefold (all P < 0.01 OB vs Chow, Fig. 3e–g), while CPT-1α was doubled by HFD consumption (P < 0.01 OB vs Chow, Fig. 3h).

### Effects of AuNP treatment

#### Anthropometric and metabolic parameters

We found that the AuNP treatments did not significantly reduce daily caloric intake by the mice (Table 2). On the contrary, the OB-EAu and OB-HAu mice consumed 8% more daily energy intake than the OB group (Table 2). Mice in the OB-EAu group demonstrated fast weight loss in the first week of treatment, however gradually regained most of the lost weight over the following 4 weeks (Additional file 1: Figure S4). Mice in the OB-LAu group had continuous weight loss after 1 week of treatment; while the body weight of the mice in the OB-HAu group mirrored that of the OB group (Additional file 1: Figure S4). The body weights of the OB-EAu and OB-LAu mice were significantly smaller than the OB mice at 15 weeks (P < 0.05, Table 2). OB-HAu mice had similar endpoint body weight as the OB mice (Table 2).

Liver weight was only significantly reduced in the OB-LAu group (P < 0.05 vs OB), while retroperitoneal fat mass was reduced in both OB-EAu and OB-LAu groups by 21% and 28%, respectively (both P < 0.05 vs OB, Table 2). The significance remains after standardization for body weight. Interestingly, epididymal fat was reduced in the OB-LAu group (P < 0.05 vs OB) but increased by 24% in the OB-EAu mice (P < 0.05 vs OB, Table 2).

During IPGTT, blood glucose levels of all AuNP-treated mice were significantly reduced to similar level as the Chow mice at 30 min (all P < 0.05 vs OB, Additional file 1: Figure S5). At 60 min, blood glucose in OB-EAu and OB-LAu groups were significantly lower than the OB group (both P < 0.05, Additional file 1: Figure S5); at 90 min, the OB-LAu group still had significantly lower blood glucose than the OB group (P < 0.05, Additional file 1: Figure S5). As such, the AUC values for all the AuNP-treated groups were significantly lower than the OB group (P < 0.01 OB-EAu and OB-LAu vs OB; P < 0.05 HAu s OB, Table 2). However, non-fasting plasma insulin levels were not significantly different between OB and AuNP-treated groups (Table 2).

The AuNP treatment was also found to improve the NEFA profile of the obese mice. NEFA levels were significantly reduced by 24% in the OB-LAu group (P < 0.05 vs OB), and 22% and 16%, respectively in the OB-EAu and OB-HAu groups however without statistical significance (Table 2). AuNP treatment had no effect on plasma triglyceride concentrations (Table 2). HDL-C were significantly elevated in all AuNP treated groups (P < 0.01 vs Chow and OB, Table 2).

Liver enzyme AST were significantly lower by 49% and 53% in the OB-LAu and OB-HAu mice respectively (both P < 0.05 vs OB, Table 2). However, ALT levels were only reduced in the OB-EAu group by 40% (Table 2). Liver triglyceride concentration was only significantly reduced in the OB-HAu group (Table 2).

#### Inflammatory and metabolic markers

In the fat issue, mRNA expression of macrophage (F4/80) and pro-inflammatory (TNFα, TLR-4) markers were significantly upregulated across all HFD + AuNP treatments compared to Chow fed mice (all P < 0.01 vs Chow, Fig. 1a–c). However, a comparison of the AuNP treated groups to the OB group showed no change at low and high doses, but a significant increase in all 3 markers by the OB-EAu mice (P < 0.01 vs OB, Fig. 1a–c).

In the liver, F4/80 was found to be reduced only in the OB-LAu group (P < 0.01 vs OB, Fig. 1d). TNFα expression was reduced in both the OB-EAu (P < 0.05 vs OB) and OB-LAu groups (P < 0.01 vs OB, Fig. 1e), while TLR-4 was downregulated across all AuNP treated groups (all P < 0.01 vs OB, Fig. 1f).

In the fat, mRNA levels of FOXO1 (P < 0.01), adiponectin (P < 0.01), PPARγ (P < 0.01), SREBP-1c (P < 0.05), FASN (P < 0.01), ATGL (P < 0.01) and CPT-1α (P < 0.01) were significantly upregulated in the OB-EAu group compared to the OB group (Fig. 2a, c–h). GLUT4 (P < 0.01, Fig. 2b) and SREBP-1c (P < 0.05, Fig. 2e) mRNA levels were reduced while CPT-1α (P < 0.05, Fig. 2h) mRNA level was increased in the OB-LAu group compared to the OB group. Adiponectin and SREBP-1c expression was downregulated in the OB-HAu group (both P < 0.01 vs OB, Fig. 2c, e).

In the liver, PEPCK, GLUT-4, SREBP-1c, FASN, and ATGL mRNA expression was significantly reduced in all AuNP-treated mice compared to the OB group (all P < 0.01 except for P < 0.05 OB-HAu vs OB for GLUT4 and SREBP-1c, Fig. 3b, c, e–g). FOXO1 and PPARγ were only downregulated in OB-EAu and OB-LAu groups (FOXO1: P < 0.01 OB-EAu vs OB, P < 0.05 OB-LAu vs OB; PPARγ: both P < 0.01 vs OB, Fig. 2a, d). CPT-1α expression was reduced in the OB-LAu group, however increased in the OB-HAu group (both P < 0.05 vs OB, Fig. 3h).

#### Distribution of gold in the organs

Trace amounts of gold were detected in all the organs of the Chow and OB groups receiving vehicle injection, which likely represents a baseline reading (Additional file 1: Table S1). All AuNP-treated mice demonstrated above baseline levels of gold in their abdominal fat, liver, spleen, kidney, heart and brain, in decreasing order (Additional file 1: Table S1). The increased levels of gold found in the abdominal fat, liver, spleen and heart reflected a dose-dependent pattern, however, significance was only observed in the high dose group compared to the other two treatment groups (Additional file 1: Table S1). Negligible amounts of gold that were detected in the brain tissues, may reflect their retention in surrounding blood vessels, as the mice were not perfused prior to organ collection.

## Discussion

In this study, mice exposed to a long term HFD diet were found to develop well known symptoms including, excessive fat accumulation and significant glucose and lipid metabolic disorders. The major findings in this study were two-fold. Firstly, it was found that daily AuNP injections in mice with existing obesity can significantly improve their lipid and glycaemic control, although a significant weight loss effect was only observed for a specific dose regime. Secondly, alterations resulting from the AuNP treatment in macrophage inflammatory responses in the liver and changes to metabolic regulators in both fat and liver tissue, are the likely underlying mechanisms. Below we discuss the two doses that were chosen based upon our previous study [23], followed by discussion of the extremely low dose treatment at the end of this section.

Long term consumption of a HFD in mice induced hyperphagia, dietary obesity and dyslipidaemia, along with subsequently developed metabolic disorders including glucose intolerance, excessive weight gain and increased fat mass, which are consistent with our previous observations [23]. Only the low dose AuNP treatment regime led to clinically significant total weight loss (12% reduction) along with fat loss effects within 5 weeks. From human clinical studies, it has been shown that weight loss greater than 5% is sufficient to improve glycaemic control and life quality in obese individuals who also have a high risk of diabetes and cardiovascular diseases [25, 26]. To date, the best weight loss treatment reported in the literature was 6.2% by Liraglutide (*Saxenda*^*®*^) where individuals were on a 1 year trial along with strict dietary controls [27]. In comparison, the AuNP treatment used in this study, induced significant levels of weight loss without changes to dietary intake. In addition, glycaemic control in the same OB-LAu treated group was nearly normalized compared to the vehicle treated obese mice. A study by BarathManiKanth et al. [28] also demonstrated that biologically synthesized 50 nm AuNPs exhibit anti-hyperglycaemic properties in diabetic mice. However, no information was provided on body weight and organ mass changes in the AuNP-treated mice. It therefore appears that AuNPs hold great promise as anti-hyperglycaemic agents, in addition to their effects on weight loss. The observed effects in the current study using the low dose AuNP regime for weight loss in mice with existing obesity (12% reduction in total body weight), was better than that achieved in our previous study which aimed to prevent weight gain due to HFD consumption (8% reduction). The effects on lowering blood glucose levels and glycaemic control during IPGTT, were however similar to those in our previous study [23].

Interestingly, the high dose regime did not exert any weight loss effect, although glycaemic control was found to be improved. This difference between the low and high doses may be due greater levels of aggregation of the high dose AuNPs following the repeated daily injections into the high ionic environment of the peritoneal fluids [29]. In addition, the adsorption of “free” soluble proteins from the peritoneal fluids onto the surfaces of the unmodified citrate-coated AuNPs can influence their ability to interact with and enter into surrounding cells and subsequently affect their function [30]. Herein, we suggest that the lower doses of AuNPs are less likely to aggregate upon IP administration, thus, maintaining their monodispersed nanoscale advantages within a physiological environment. Further studies are therefore warranted to define the physical changes that occur to AuNPs when inserted into the body. Such studies are currently being undertaken and are revealing interesting new phenomena [31].

Inflammatory processes, especially those involving the pro-inflammatory cytokine TNFα and its signalling pathway, play a key role in insulin resistance and glucose intolerance [32]. The accumulation of excess lipids in the fat tissue is known to attract the migration of monocytes from the blood stream into the adipose tissue to form resident adipose tissue macrophages which then produce the protein cytokine, TNFα [33]. The marker F4/80 is used to identify active macrophages which possess phagocytic properties and produce significant amounts of pro-inflammatory cytokines. Following the same two low and high dose ip injection regimes as our previous study [23], the current data revealed that in the fat tissue neither F4/80 nor the other inflammatory markers screened were affected, suggesting the AuNPs did not change macrophage activity, or recruit more monocyte cells into the fat tissues. This is distinct to our findings in our previous study, where AuNP treatment and HFD consumption were begun simultaneously. In the former study, expression of TNFα mRNA was reduced even without changing the macrophage number [23]. This may suggest that AuNPs can prevent the overexpression of TNFα mRNA, while consuming a HFD diet, but not downregulate it.

Lipid accumulation in hepatocytes correlates with an increase in adiposity leading to liver steatosis, commonly accompanied with long term HFD-induced obesity [34]. Similar to that seen in adipocytes, steatosis also induces low grade inflammatory responses and activation of Kupffer cells (liver resident macrophages) in the liver of obese mice [18, 19]. The heterogeneity of Kupffer cells have strong cytokine-producing capacity [35], as shown in our current study where liver F4/80 as well as inflammatory markers including TNFα and TLR-4 were all significantly upregulated in vehicle-treated obese mice, indicating an activation of liver Kupffer cells, consistent with hepatic steatosis. Interestingly, upon AuNP treatment, the downregulation of inflammatory markers TNFα and TLR-4, mirrored the change in F4/80, which is consistent with our previous study [23], which may also be a contributing factor to the improved glycaemic control during IPGTT.

Previously, we have shown that improved substrate metabolic markers following AuNP treatment, occurs via interactions between macrophage cells and fat cells [23]. Although in the current study the pro-inflammatory property of the macrophages in the fat tissue did not appear to be affected by low and high doses of AuNP treatment, expression levels of several metabolic markers were altered in the OB-LAu and OB-HAu mice. In the OB-LAu mice, insulin responsive glucose transporter GLUT4 and de novo lipogenesis regulator SREBP-1c were downregulated in the mice treated with low AuNP dose, suggesting reduced glucose uptake into the cells for de novo lipid synthesis for fat storage. In addition, expression of the marker for fatty acid oxidation, CPT-1α was doubled in the OB-LAu mice. CPT-1α is the rate limiting step for the transportation of long-chain fatty acid into the mitochondria for β-oxidation [36], upregulation of which may encourage the breakdown of more fat storage, leading to reduced fat mass, as was observed in this study. However, CPT-1α was not increased in the OB-HAu mice albeit reduced GLUT4 and SREBP-1c levels, which may account for their excessive adiposity when daily energy intake was higher than the OB mice. In the OB-HAu mice, the suppressor of gluconeogenesis FOXO1 [37] was reduced, which may result in increased glucose conversion from fat or protein to raise blood glucose levels. In addition, reduced adiponectin may also result in insulin resistance in insulin sensitivity organs, including liver and fat. This may partly explain why the glycaemic control in the OB-HAu mice was not as good as the OB-LAu mice.

Liver also plays a critical role in both systemic glucose and lipid metabolism. Down regulation of the PEPCK gene can diminish gluconeogenesis and its expression is commonly up-regulated in type 2 diabetes, with FOXO1 known to inhibit PEPCK expression to counteract its effect [38]. PPARγ is another insulin sensing and glucose homeostatic regular which also involves GLUT4, the insulin dependent glucose transporter [39]. In the vehicle treated-obese mice, all these genes were upregulated, whereas AuNP treatments using low and high doses either normalized or significantly down regulated their expression. In addition, excess lipid accumulation in the liver can also contribute to insulin resistance [40, 41]. Here, in the obese mice, liver SREBP-1c and FASN which are crucial for lipogenesis and fatty acid biogenesis respectively were also significantly upregulated in the face of increased liver triglyceride concentration, which can’t be reversed by the adaptive upregulation of ATGL and CPT-1α, which regulates liver triglyceride turnover and free fatty acid β-oxidation [42]. Again, all the above-mentioned abnormal expression of lipid metabolic markers were also normalized or improved in the OB-LAu and OB-HAu mice.

Long term chronic energy surplus often contributes to dyslipidaemia commonly associated with metabolic disorders such as, insulin resistance and cardiovascular disease risk [43, 44]. HDL-C is known for this anti-inflammatory effect and protection against atherosclerosis, whose level is normally reduced in individuals with metabolic disorders [45]. Its mimetic has also been shown to improve hepatic insulin resistance by reducing inflammatory responses [46]. AuNP treatment in this study showed a significant augmentation of HDL-C in mice with existing obesity, and reduced blood lipid levels. As such, AuNP treatment may not just be applicable in diabetes, but also dyslipidaemia.

The most interesting observation in this study is the extremely low dose group, which we discuss here separately. There was initial rapid weight loss observed in the first week, however sustained growth and weight gain soon followed. The catch-up weight gain likely reflects the adaptation to rapid weight loss, where the body tries to restore normal body weight set-point. In this study, this was achieved by over-consumption of 18% more energy than the vehicle treated obese mice. The changes in metabolic markers in the fat tissue of these mice also well reflect the process of excessive influx of dietary lipids, including an increase in macrophages and inflammatory profile, increased de novo fat synthesis, lipolysis and β-oxidation, which are normally observed in mice undergoing weight gain [47, 48]. However, a fat re-distribution was also observed, with reduced retroperitoneal fat mass but increased epididymal fat mass. It is believed that visceral fat is principally responsible for the pro-inflammatory status and related metabolic disorders in obesity, whereas epididymal fat is involved in reproductive functions [49]. As such, it is reasonable to understand why the OB-EAu mice were gaining weight, while their metabolic profile was better than the vehicle treated obese mice. However, understanding why this particular dose of AuNPs can induce fat redistribution still requires further investigation. In addition, even with a very low dose, AuNPs still showed marked benefits by improved glycaemic control during IPGTT and increase blood HDL-C levels, which may contribute to their effects in the liver to reduce inflammatory responses and improve all the metabolic markers measured here. As such, although the extremely low AuNP dose is not potent to induce sustained weight loss effects, it can still be considered for the treatment of glucose metabolic disorders. In addition, coupled with a low fat diet, this dose regime may potentially cause satisfactory weight loss effects. This also warrants further investigation in follow up studies.

Due to the size range, 20–30 nm of the AuNPs used in this study, it is estimated that they would preferentially be eliminated via the reticuloendothelial system such as liver and spleen, with limited excretion through the renal system via the kidneys, consistent with previous animal studies [50, 51]. Highest Au levels were found in the abdominal fat suggesting that injected AuNPs were absorbed directly from the peritoneal cavity into the surrounding adipose tissue and then distributed to other tissue via the circulation [24, 50]. Most importantly, AuNP-treatment did not cause measurable liver toxicity or cell damage, reflected by unchanged levels of the two enzymes AST and ALT, the increase of which normally indicate hepatocyte damage. However, the accumulation of AuNPs into specific tissues and organs still raise concerns for the use of high doses AuNP, even though there were some health benefits in the obese mice. Oral intake may ameliorate such over accumulation, as the gut can selectively adsorb materials. Again, such issues require further investigation.

The precise mechanism of how the citrate stabilised AuNPs induce these cellular changes, that in turn result in changes to whole body physiology, still remains unclear. There is now clear evidence that the local environment and route of administration of the nanoparticles play a critical role in their subsequent cellular interactions within the biological system. This has been reviewed in [31, 52] which point to the key role of the “corona” that forms around the particles via the binding of biomolecules present in the extracellular milieu. These biomolecules include, but are not limited to, proteins, lipids, DNA, microRNA and sugars. They form a specific signature or fingerprint on the nanoparticle that will then dictate the nanoparticles’ binding to and recognition by target or non-target cells. This will in turn dictate the cellular response and internal localisation of the particle. Similarly, the size and shape of the particles are also known to influence the nanoparticles’ route of internalisation (e.g. receptor-mediated endocytosis or pinocytosis or phagocytosis or other) along with their subsequent intracellular fate [53]. As such, the initial nanoparticle synthesis itself ultimately dictates the downstream in vivo outcomes, as shown in a recent study by Rodriguez-Lorenzo et al. [54].

In vivo, typical proteins that attach to AuNPs include albumin, immunoglobulins, fibrinogen, and apolipoproteins [55]. It has been speculated that conformational change of these proteins may also affect protein–protein interactions, which eventually affect the downstream cellular signalling and DNA transcription [55]. We can speculate that in our study, apolipoproteins or high density lipoproteins (HDL) within the corona play a role, as HDL has been shown to have potent anti-inflammatory properties [56], yet it can also induce a pro-inflammatory response in macrophages [57]. We have shown in our previous study that AuNPs can induce an inflammatory response if incubated with macrophages, which was however not observed when we performed in vitro studies co-culturing macrophages with adipocytes [23]. We also found that co-culturing was necessary for AuNPs to modulate metabolic markers, suggesting an interaction between adipocytes and macrophages to improve substrate metabolism [23]. As such, more detailed investigations are needed to further elucidate details of the AuNP and cellular interactions that underpin the systemic physiological changes observed in our current study.

Regardless of how AuNPs interact with body fluid components, the direct biological outcome is reduced macrophage number and their related pro-inflammatory cytokines, as consistently shown here and in our previous studies [23, 24]. There are currently 6 groups of anti-diabetic medications available on the market to manage blood glucose level in patients with type 2 diabetes. However, none of these have the same potent effect as AuNPs to suppress the inflammatory response by inhibiting macrophages, which is the fundamental mechanism driving the development of glucose intolerance and thus is identified as the new target for development of anti-diabetic medications [58]. Our studies in fact confirm that macrophage cells are a plausible target for this purpose. With further development of our AuNPs or similar, this strategy has the potential to be added to the current double or triple therapy with metformin.

## Conclusions

In conclusion, our results suggest that reduced local inflammation via regulation of macrophage recruitment and activity in both adipose tissue and the liver by AuNPs, are one of the key mechanisms for the effects observed, resulting in improved lipid and glucose metabolism and weight loss, in mice with existing obesity. The therapeutic value of AuNPs and their anti-obesity and anti-diabetic properties is further confirmed by the positive outcomes observed in our studies using mice with existing obesity. This gives increased promise for the future development of a novel AuNP treatment strategy for use with obese and diabetic populations. Biotechnological advancements in therapeutic and prophylactic treatments are also served by our ability to have a detailed understanding of the interaction between such nanosized materials and cells in the human body and within living systems.

## Methods

### Synthesis and characterization of spherical AuNPs

Spherical AuNPs with a hydrodynamic diameter between 16 and 147 nm can be synthesized when the trisodium citrate-to-gold ratio was varied via the citrate sol method [59, 60]. The spherical 20–30 nm AuNPs used in this study were synthesized as previously described [24, 61]. The as-synthesized AuNPs were centrifuge-purified in batches of 50 mL at 5000 rpm, 4 °C for 30 min to remove excess citrate from solvent and AuNPs were resuspended in water for animal studies.

Various advance material techniques including dynamic light scattering, high resolution-scanning electron microscopy (HR-SEM), and UV–VIS were employed to characterize the size, shape, zeta potential, and optical properties of as-synthesized AuNPs. The hydrodynamic diameter of gold colloids was determined by Brookhaven ZetaPALS (Holtsville, NY, USA) using a quartz cells with 1 cm path length. Zeta potential of AuNPs in water was measured using Malvern NanosizerZS^®^ instrument (Malvern, Worcestershire, UK) using a 2 mm, ~ 8–10 attenuators, clear disposal zeta cell. Both measurements were performed in water suspension in triplicate at 20 cycles per run. Results were presented as mean ± S.E.M. All HR-SEM images were obtained in either bright-field or backscatter mode using LEO Supra 55VP SEM (Zeiss, Jena, Germany) with electron backscatter diffraction (EBSD). The HR-SEM was performed under system vacuum pressure greater than 3.4 × 10^6^ Torr at high current of 20 kV. In order to spread the AuNPs evenly onto the SiO2 wafer for HR-SEM, an amino-functional silane, (3-aminopropyl)-triethoxysilane was used as an adhesion or anchoring agent for the AuNPs as previously described [62]. This allow determine size and shape by immobilization of monolayer AuNPs on silicon substrate. The UV–VIS spectra were acquired with a HP 8453 spectrophotometer (Agilent Technologies Deutschland GmbH, Waldbronn, Germany) using a quartz cells with 1 cm path length. The spectra were obtained over the range of 190–1100 nm where optical characteristic surface plasmon resonance band and peaks were determined.

### Animal experiments

All procedures in this study were approved by the Animal Care and Ethics Committee at the University of Technology Sydney (ACEC#2011-403A) in accordance with the Australian Code for the Care and Use of Animals for Scientific Purposes by the Australian, National Health and Medical Research Council.

Male C57Bl/6 mice (7 weeks, Animal Resource Centre, WA, Australia) were housed at 20 ± 2 °C on a 12:12 h light/dark cycle. Mice were fed standard rodent chow (11 kJ/g, 14% fat, Gordon’s Specialty Stockfeeds, NSW, Australia) with ad libitum access to water during the acclimatization period. The mice were divided into 5 groups with equal body weights (n = 16): 1 group was fed standard rodent chow while 4 other groups were fed a HFD (20 kJ/g, 43% fat, Specialty Feeds, WA, Australia [23]) for 10 weeks to induce obesity. From week 11, the chow-fed (Chow) group and one of the HFD-induced obesity (OB) groups received 5 weeks of daily vehicle (water) intraperitoneal (ip) injection. The remaining three HFD-fed groups received daily ip injection of either low dose AuNP (OB-LAu, 0.785 μg/g/day, ip.), or high dose AuNP (OB-HAu, 7.85 μg/g/day, ip.) as we have previously published [23] with the third group receiving an extremely low dose AuNP (OB-EAu, 0.0785 μg/g/day, ip.) for 5 weeks. The maintenance diets were not changes during the treatment. Body weights and energy consumption (kJ/mouse/24 h) was measured fortnightly before the treatment and weekly during the 5 weeks of treatment.

At week 14, mice (n = 8) were fasted for 5 h after which time an IPGTT was performed and the AUC of glucose changes was calculated for each animal as described in our previous study [63].

At the end point, the mice were euthanized by sodium thiopental (0.1 mg/g, IP, Abbott Diagnostics, Kurnell, NSW, Australia). Blood was collected via cardiac puncture and plasma was separated and stored at − 20 °C for bioassay analyses. The organs including heart, spleen, kidneys, liver, and abdominal fats were harvested and weighed, followed by fixation in 10% formalin or snap frozen in liquid nitrogen.

### Bioassays

Plasma and liver tissue lysates were used for biochemical assays to determine concentration of NEFA and triglycerides as described previously [63]. Plasma concentrations of insulin, AST and ALT in were measured according to the manufacturer’s protocol [23]. Plasma HDL-C concentration was measured using the Cholesterol HDL-C kit (D00127, Dialab Ltd., Vienna, Austria) according to manufacturer’s protocol.

### Real time-PCR

The mRNA was extracted from the fat and liver tissue as previously described [23]. The purified total RNA at 200 ng/µL was used for cDNA synthesis using M-MLV Reverse Transcriptase, RNase H Minus, Point Mutant Kit (Promega, WI, USA). mRNA expression of target genes, including markers of macrophage (CD68 and F4/80), inflammation (TNFα, TLR-4), glucose metabolism (adiponectin, GLUT-4, FOX-O1, PEPCK, PPARγ), and lipid metabolism (ATGL, CPT-1α, SREBP-1c, FASN) were measured using manufacture pre-optimised TaqMan^®^ or SYBR Green II assays (Thermo Fisher Scientific, CA, USA).

### ICP-MS analysis

The fat, liver, spleen, kidney, heart, lung and brain tissues were analysed for the concentration of elemental gold using ICP-MS as described in our previous publication [23, 64]. Briefly, freeze-dried samples (~ 0.05 g) were digested with aqua regia and hydrogen peroxide (1:2:1 HNO_3_, HCl, and H_2_0_2_ respectively, ultrapure Baseline Seastar reagents supplied by Choice Analytical, NSW, Australia), before a 1:1 dilution in ultrapure water. The samples were analysed on an Agilent Technologies 7500cx ICP-MS (Agilent Technologies, VIC, Australia) equipped with a MicroMist concentric nebuliser (Glass Expansion, VIC, Australia) and a Scott type double pass spray chamber cooled to +2 °C. The ICP-MS extraction lens conditions were selected to maximise the sensitivity of a tune solution containing 1 ng mL^−1^ Li, Co, Yb, Ce, and Tl in 1% HNO_3_/HCl, with helium added into the octopole reaction cell to reduce interferences. Calibration curves were matrix matched with a concentration range (0–1000 ng mL^−1^) and constructed using a certified Au calibration standard (Choice Analytical, NSW, Australia). The results were analysed using the Agilent Technologies MassHunter Workstation software.

## Additional file

**Additional file 1.** Additional Data providing a Tabular summary of the distribution of AuNPs in the various body organs; Characterisation of the synthesised AuNPs including dynamic light scattering, UV-Vis absorption spectra and SEM images; Graphical summaries of the effects of AuNP treatments on mice body weights and glucose metabolism.

## Abbreviations

ATGL: adipose triglyceride lipase
ATM: adipose resident macrophages
AuNP: gold nanoparticle
Chow-C: chow-fed mice treated with vehicle
CPT: carnitine palmitoyltransferase
HDL: high density lipoprotein
HFD: high fat diet
IP: intraperitoneal
IPGTT: intraperitoneal glucose tolerance test
OB-C: obese mice treated with vehicle
OB-EAu: obese mice treated with an extremely low dose of AuNP
OB-HAu: obese mice treated with a high dose of AuNP
OB-LAu: obese mice treated with a low dose of AuNP
PPAR: peroxisome proliferator-activated receptor
SREBP: sterol regulatory element-binding proteins
TNF: tumour necrosis factor
