# Supplementary material for: Gold nanoparticles as cell regulators: beneficial effects of gold nanoparticles on the metabolic profile of mice with pre-existing obesity
Source: J Nanobiotechnology. 2018 Nov 3;16:88. doi: 10.1186/s12951-018-0414-6 (PMC6215354; doi:10.1186/s12951-018-0414-6)
Supplement: Supplementary file 1 — Additional file 1. Additional Data providing a Tabular summary of the distribution of AuNPs in the various body organs; Characterisation of the synthesised AuNPs including dynamic light scattering, UV-Vis absorption spectra and SEM images; Graphical summaries of the effects of AuNP treatments on mice body weights and glucose metabolism. [file 12951_2018_414_MOESM1_ESM.docx]

**Additional data**

Table S1: Concentration of gold in the organs

| µg/g  tissue | Chow-C | OB-C | OB-EAu | OB-LAu | OB-HAu |
| --- | --- | --- | --- | --- | --- |
| Fat | 0.22 ± 0.01 | 1.89 ± 0.49 | 36.91 ± 14.61 | 750 ± 187 | 10692 ± 2207‡ |
| Liver | 0.42 ± 0.05 | 0.54 ± 0.14 | 14.83 ± 3.52 | 624 ± 311 | 3052 ± 1959*† |
| Spleen | 0.72 ± 0.30 | 0.69 ± 0.28 | 3.28 ± 0.65 | 21.41 ± 6.63 | 236.1 ± 113.6‡ |
| Kidney | 0.16 ± 0.07 | 0.07 ± 0.01 | 0.16 ± 0.02 | 0.93 ± 0.17 | 2.73 ± 0.95‡ |
| Heart | 0.01 ± 0.01 | 0.01 ± 0.00 | 0.06 ± 0.01 | 0.42 ± 0.13 | 2.64 ± 1.25‡ |
| Brain | 0.12 ± 0.04 | 0.05 ± 0.01 | 0.08 ± 0.01 | 0.09 ± 0.01 | 0.28 ± 0.14† |

Results are expressed as mean ± S.E.M. Data were analysed by one-way ANOVA followed by post hoc Bonferroni test.* P < 0.05 *vs.* Chow; † P < 0.05 *vs.* OB; ‡ P < 0.05 *vs.* all the other groups. n=3.


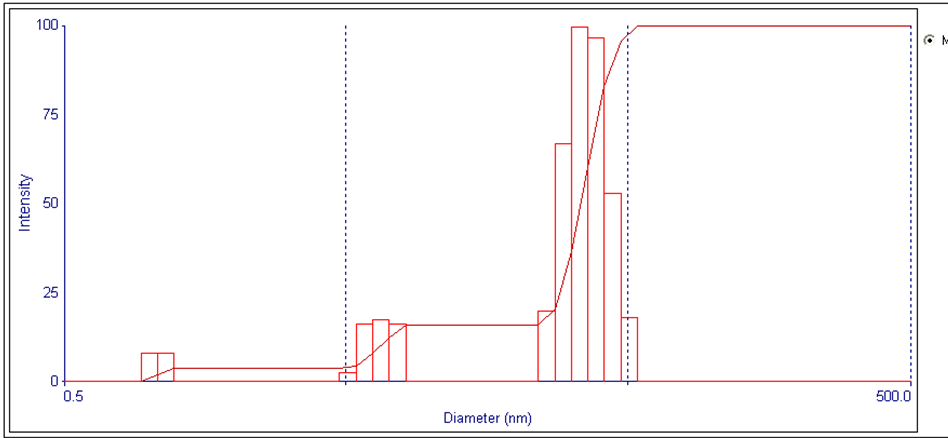


**Figure S1**

Dynamic light scattering measurement of as-synthesized AuNPs showing a narrow distribution of particle size between 20–30 nm and mean hydrodynamic diameter of 27.3±0.5 nm as determined by Brookhaven ZetaPALS.

Figure S2

UV-VIS absorption spectra showed a slight shift in surface plasmon resonance peaks for as-synthesized AuNPs in water from 520 nm to 518 nm for AuNPs resuspended in water after centrifuge purification at 5000 rpm, 4°C for 30 mins to remove excess citrate capping ions.


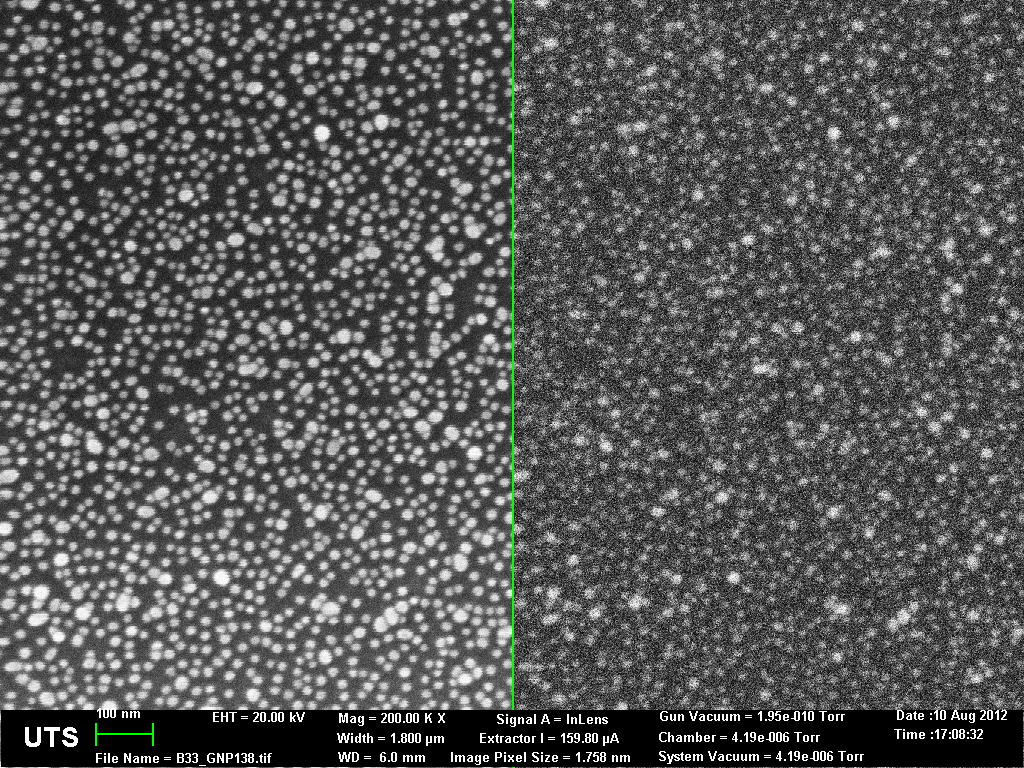


(A)


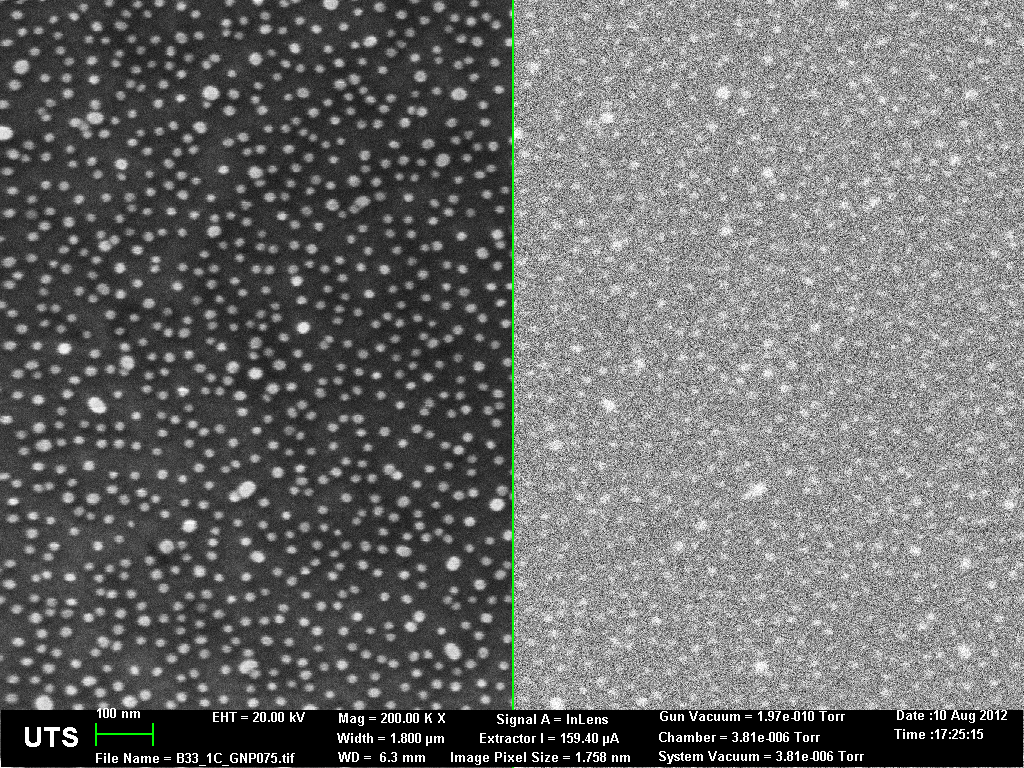


(B)

Figure S3

Representative brightfield mode (left panel) and electron backscatter diffraction mode (right panel) HR-SEM images at 200,000x magnification of as synthesized AuNPs immobilized on SiO­_2_ wafer using functional silane before (A) and after centrifuge-purified (B) dispersed in water. More than 60–100 particles were manually measured for particle core size estimation. Scale bar = 100 nm.

Figure S4. Effects of HFD and AuNP treatment on body weight in male mice. Body weight (B) in Chow (□ solid blue line), OB (◼ solid red line), OB-EAu (⚫ dotted green line), OB-LAu (⯅ dotted purple line), and OB-HAu (⧫ dotted orange line). Results are expressed as mean ± S.E.M. Data were analysed by one-way ANOVA followed by post hoc Bonferroni test. γ P < 0.05 Chow *vs.* OB-EAu & OB-LAu; * P < 0.05 Chow *vs.* all other groups; λ P < 0.05 Chow *vs.* OB-LAu; ϕ P < 0.05 Chow *vs.* OB-HAu; ‡ P < 0.05 OB *vs.* OB-EAu & OB-LAu; τ P < 0.05 Chow *vs.* OB & OB-EAu; ξ P < 0.05 OB *vs.* OB-EAu & OB-HAu; ε P < 0.05 OB *vs.* OB-EAu; ζ P < 0.05 OB *vs.* OB-LAu; n=13–16.

Figure S5. Effect of HFD and AuNP treatment on glucose metabolism. Blood glucose levels during intraperitoneal glucose tolerance test (IPGTT) in Chow (□ solid blue line), OB (◼ solid red line), OB-EAu (⚫ dotted green line), OB-LAu (⯅ dotted purple line), and OB-HAu (⧫ dotted orange line) mice at week 14. Results are expressed as mean ± S.E.M. Data was analysed by one-way ANOVA with repeat measures followed by post hoc Bonferroni test. ‡ P< 0.05 Chow *vs.* all other 4 groups at 15min; γ P< 0.05 OB *vs.* all other 4 groups at 30mins; δ P< 0.05, Chow *vs.* OB & OB-HAu, OB *vs.* OB-EAu & OB-LAu at 60mins; θ P< 0.05, Chow *vs.* OB & OB-HAu, OB *vs.* OB-LAu at 90mins.
